# Social connectedness and dementia prevention: Pilot of the APPLE-Tree video-call intervention during the Covid-19 pandemic

**DOI:** 10.1177/14713012211014382

**Published:** 2021-04-29

**Authors:** Claudia Cooper, Hassan Mansour, Christine Carter, Penny Rapaport, Sarah Morgan-Trimmer, Natalie L Marchant, Michaela Poppe, Paul Higgs, Janine Brierley, Noa Solomon, Jessica Budgett, Megan Bird, Kate Walters, Julie Barber, Jennifer Wenborn, Iain A Lang, Jonathan Huntley, Karen Ritchie, Helen C Kales, Henry Brodaty, Elisa Aguirre, Anna Betz, Marina Palomo

**Affiliations:** 4919 UCL, London, UK; 9747 Camden and Islington NHS Foundation Trust, London, UK; 4919 UCL, London, UK; 3286 University of Exeter, Exeter, Devon, UK; 66046 UCL, London, UK; 4919 UCL, London, UK, 5098 North East London NHS Foundation Trust (NELFT), Ilford, UK; 3286 University of Exeter, Exeter, UK; 4919 UCL, London, UK; 27102 INSERM, Paris, France; 8789 University of California Davis, Davis, CA, USA; 7800 University of New South Wales, Sydney, New South Wales, Australia; 4919 UCL, London, UK; 5098 North East London NHS Foundation Trust (NELFT), London, UK; 9747 Camden and Islington NHS Foundation Trust, London, UK

**Keywords:** cognition, mild cognitive impairment, eHealth, remote, internet, subjective cognitive decline, older adult

## Abstract

**Background and Objectives:**

The Covid-19 pandemic reduced access to social activities and routine health care that are central to dementia prevention. We developed a group-based, video-call, cognitive well-being intervention; and investigated its acceptability and feasibility; exploring through participants’ accounts how the intervention was experienced and used in the pandemic context.

**Research Design and Method:**

We recruited adults aged 60+ years with memory concerns (without dementia). Participants completed baseline assessments and qualitative interviews/focus groups before and after the 10-week intervention. Qualitative interview data and facilitator notes were integrated in a thematic analysis.

**Results:**

12/17 participants approached completed baseline assessments, attended 100/120 (83.3%) intervention sessions and met 140/170 (82.4%) of goals set. Most had not used video calling before. In the thematic analysis, our overarching theme was *social connectedness*. Three sub-themes were as follows: **Retaining independence and social connectedness**: social connectedness could not be at the expense of independence; **Adapting social connectedness in the pandemic**: participants strived to compensate for previous social connectedness as the pandemic reduced support networks; **Managing social connections within and through the intervention:** although there were tensions, for example, between sharing of achievements feeling supportive and competitive, participants engaged with various lifestyle changes; social connections supported group attendance and implementation of lifestyle changes.

**Discussion and Implications:**

Our intervention was acceptable and feasible to deliver by group video-call. We argue that dementia prevention is both an individual and societal concern. For more vulnerable populations, messages that lifestyle change can help memory should be communicated alongside supportive, relational approaches to enabling lifestyle changes.

## Introduction

Dementia and its prevention constitute one of the greatest health and social challenges of our time (Prince et al., 2013). The global Covid-19 pandemic has exacerbated most modifiable dementia risk factors – including cardio-metabolic disease, physical inactivity, social isolation, mental illness and alcohol consumption (Livingston et al., 2020). Covid-related social distancing measures reduce opportunities for activities, socialising and exercise (Heid et al., 2020), and non-Covid health and social care availability has also been affected by the pandemic (Giebel & Cooper, 2020).

The pandemic has, at least to some extent, shifted responsibility for lifestyle choices, such as social encounters, from individuals to society. This may influence already controversial debates around how responsibility for dementia prevention is shared across individuals and society. Half of over 65s in the United Kingdom fear dementia more than any other condition (Monitor, 2019), so it is unsurprising that interventions discussing dementia risk are anxiety-provoking. We have previously described how living with memory problems without dementia may be conceptualised as liminal, between dementia and wellness, and that individuals may experience the burden of responsibility for managing dementia risk, without access to the help that may follow a definitive diagnosis (Poppe et al., 2020). Libert et al. (2019) explore individualistic attitudes around dementia prevention. He suggests that adopting lifestyle change for dementia prevention can be viewed as an emotional, as well as practical response to fear of dementia: as emotional distancing from dementia, a condition associated with ‘ageing without agency’.

Resilience is defined as the process of ‘bouncing back’ from difficult experiences (MacLeod et al., 2016). In this study, we seek to support older people experiencing memory concerns to adopt lifestyle changes that reduce dementia risks; put another way, we seek to enable a resilient response to the often anxiety-provoking experience of developing memory concerns. The older population have exhibited high resilience levels in studies that interviewed relatively healthy older populations, including cohorts recruited early in the pandemic, about their reactions to stressful events (Knepple Carney et al., 2020). Yet resilience is an interaction between individuals and the social environment and should not be construed as an individual achievement (Kok et al., 2018). Previous work critiques the positioning of all older people as consumers of lifestyle choices enabling the ‘third age’, defined by Laslett as ‘a period of agentic self-fulfilment’ (Gilleard & Higgs, 1998). Not all older people are equally able to exhibit resilience, leading to new social divisions. An emphasis on agency has the effect of making individuals responsible for their own health whether or not this is possible; dementia prevention must also be viewed as a societal concern (Higgs & Gleard, 2015).

In reality, while there is evidence that risk factor modification reduces dementia risk (e.g.Ngandu et al., 2015), dementia prevention efforts, whether targeted at individuals or society, are in their infancy. Certainly, no currently available interventions, with proven efficacy, are scalable to whole populations (Brug, 2008). Rapid expansion in eHealth interventions due to social distancing will influence future dementia prevention, and eHealth dementia prevention interventions targeted at the general, older population are under evaluation (Heffernan et al., 2018).

We coproduced the APPLE-Tree *(Active Prevention in People at risk of dementia through Lifestyle, bEhaviour change and Technology to build REsiliEnce)* intervention, specifically for people with memory concerns without dementia, who are at increased dementia risk (Mitchell & Shiri-Feshki, 2008). In response to the pandemic, we adapted our face-to-face group programme, which is based on current evidence (Whitty et al., 2020), to remote delivery. While remote interventions can have excellent reach and cost-effectiveness, they may be challenging for people with memory concerns to access and can compound socio-economic inequalities (Jaffe et al., 2020). They could also engender shifts towards individualistic approaches to dementia prevention.

To our knowledge, this is the first study to explore how older people with memory concerns experienced and used a video-call, group-based cognitive well-being intervention, which also included individual phone calls to participants to support goal-setting. Our research objective was to investigate how acceptable and feasible the intervention was to deliver in practice, in the context of the pandemic. We were interested in exploring through participants’ accounts how the intervention was experienced and used in the pandemic context. Our research questions were thus:

1. How acceptable and feasible was the intervention to deliver in practice?
2. How was the intervention experienced and used in the pandemic context?

## Methods

### Design

We conducted a pre-/post-test single group, pilot study of a remote (group-based video-call) cognitive well-being intervention, APPLE-Tree; with a multiple-method exploratory design.

### Ethical approval and trial registration

London-Camden and Kings Cross National Research Ethics Committee approved the study (20/LO/0034); and we registered the protocol (ISRCTN17325135) (Cooper et al., 2019).

### Intervention development

We coproduced APPLE-Tree with older people with memory concerns, their family members, health practitioners and researchers, informed by the behaviour change framework (Michie et al., 2011). Six coproduction workshops involved academic professionals, healthcare practitioners, third sector workers and experts by experience in the intervention target domains: nutrition, physical exercise, physical health, social engagement, cognitive stimulation, sleep and mental well-being. We used the groups’ expertise, informed by current evidence and existing interventions (Hassan et al., 2018; Livingston et al., 2019) to produce participant workbooks and facilitator manuals to guide the planned, structured sessions.

We originally initially designed 10, 1.5–2 h face-to-face groups for 10–12 participants, led by two facilitators, with a refreshment break when facilitators would support participants to set goals. In April 2020, our coproduction group held remote workshops, to consider how the intervention might be adapted to remote delivery and to account for pandemic-related social changes. We developed a remote version that was similar in content and intended mechanisms of action to the planned face-to-face format, for delivery on Zoom™. We added facilitator prompts acknowledging that lifestyle change may be more challenging and need adapting, in the pandemic context.

### Intervention structure

Before the first session, participants received a non-perishable food delivery (e.g. olive oil and frozen vegetables) costing approximately £18, to support home cooking; a step-counting watch; the session workbook and a structured booklet for recording goals and progress.

Each week, participants were invited to

**Figure 1.**
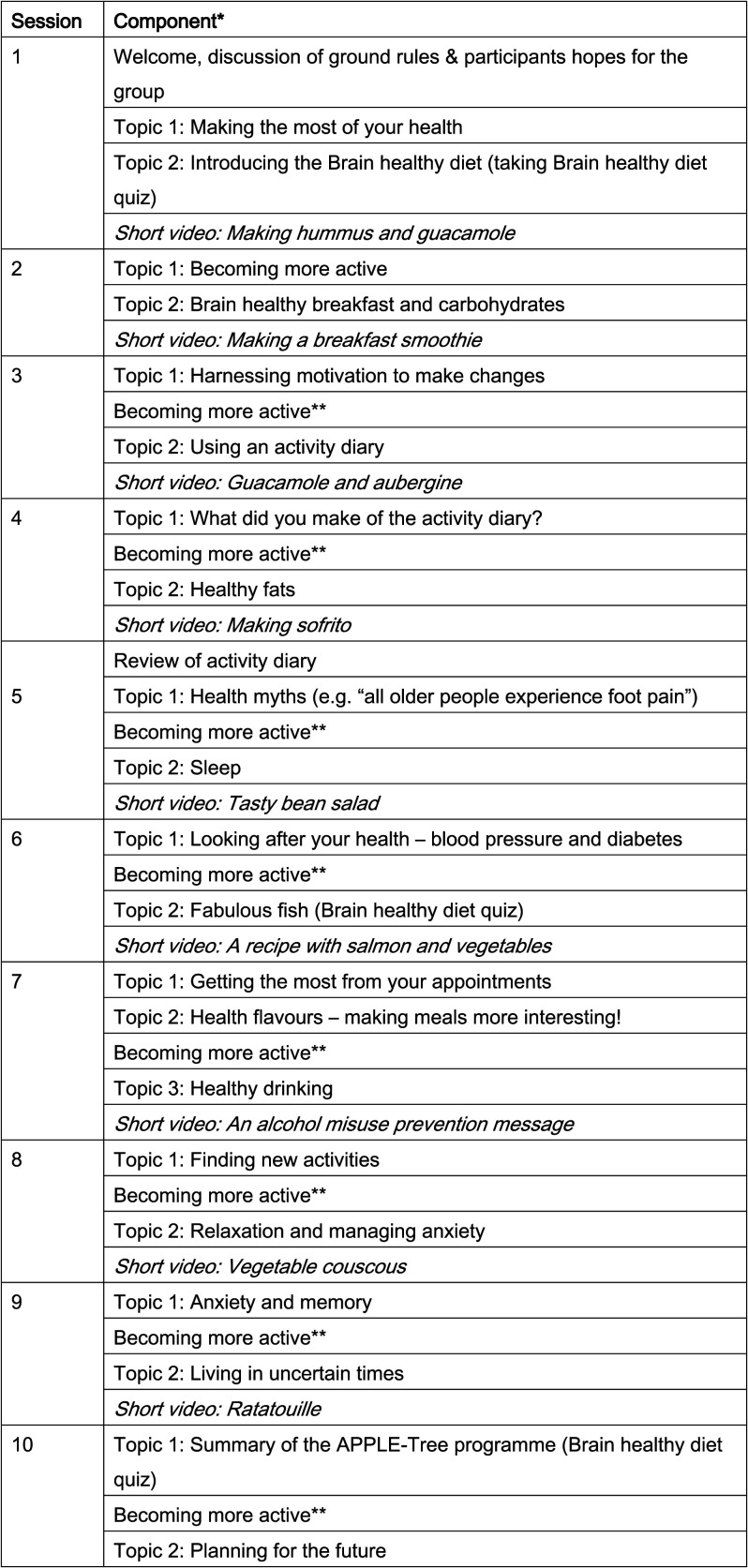
APPLE-Tree sessions with intervention components listed. *Sessions (2+) begin by reviewing goals achieved and end discussing new goals. ** participants discussed new forms of exercise– sharing ideas or selecting a short video from a menu of high- and low-impact options.

- A one-hour group video-call (run as 2 smaller groups a couple of hours apart, with ≤6 participants, with 2 facilitators and 1 helper): discussing ways to promote cognitive well-being (related to intervention targets; Figure 1), including short video cookery demonstrations, which participants were encouraged to try and bring to ‘tea break’. Sessions were fully manualised. Participants were encouraged to share photos and short videos of lifestyle changes and activities tried.
- A half-hour ‘tea break’ with all participants together on one video-call (i.e. ≤12 participants). Sessions were unstructured; facilitators encouraged discussion of how participants were implementing the well-being–promoting lifestyle changes. Whereas the structured groups were kept small to enable focussed discussions, the tea break was a larger group intended as a less formal space.
- A phone call (up to 30 min) with one facilitator. Participants were encouraged to set new and revise existing goals, recording progress in their goal-setting booklet. Possible goal areas were as follows: nutrition (participants set bronze, silver and gold goals, to increase their Mediterranean Diet Score (MDS) score by 1, 2 then 3 points from baseline); physical activity (to increase activity, which could be measured by recording highest daily step count, using provided step-counting watches); engaging with life (planning activities to move nearer to the life they want to live); connecting with others and health (e.g. planning blood pressure or hearing checks, staying hydrated and reducing alcohol intake and smoking and increasing the use of mindfulness and sleep hygiene).

### Training and supervision

We trained two facilitators with experience of working with people with dementia: a UCL-employed psychology graduate (HM) and a worker from the voluntary organisation from which we recruited participants. They role-played sessions with the research team, which PR/CCo formally assessed for adherence to the manual and skill prior to delivery. They received weekly group supervision with a clinical psychologist (PR) and/or psychiatrist (CCo), troubleshooting barriers to delivery and exploring engagement strategies. PR/CCo was available for support between supervision meetings. We trained facilitators on adaptations to remote delivery, for example, how to introduce video calling to new users and use of the mute facility to ensure smooth running of the groups. In addition to the two facilitators, CCa joined groups as a helper to support participants with their internet connectivity if required and ensure group continuity if there were technical problems.

### Sampling and participants

We recruited older adults with mild cognitive impairment (MCI) or subjective cognitive decline (SCD) from one-third sector partner organisation and one London NHS Trust. The partner organisation advertised the sessions in their newsletter and at events; and staff sought agreement of interested members to be approached by researchers. We also advertised groups on social media. NHS staff approached patients at the NHS Trust. We included adults aged 60+ years, who self-determined that they were sufficiently proficient in English to participate in groups, without a known dementia diagnosis and with capacity to consent to participation, as judged by the research team after appropriate training. Having internet access or computer proficiency was not inclusion criteria. We excluded people with a terminal condition, considered to be in the last 6 months of life.

As part of screening, participants completed

*The Quick MCI* has good psychometric properties for distinguishing normal cognition from MCI/dementia; we excluded people scoring under accepted age and education-adjusted cut-points that indicated dementia (O’Caoimh et al., 2017). We included participants scoring in the range of subjective cognitive impairment (SCD) (>62; total possible score range 0–100) (O’Caoimh et al., 2012) where respondents gave an affirmative response to the question: ‘has your memory deteriorated in the last 5 years?’; and to either the question ‘Are you concerned about this?’ or ‘Is your memory persistently bad?’ This approach is adapted from published measures of SCD (Jessen et al., 2020).

*The Functional Assessment Questionnaire scale* (Pfeffer et al., 1982), measuring dependency for activities of daily living. We excluded participants scoring 9+ (indicating possible functional impairment; score range 0–30, with 30 indicating greatest dependency) unless impairment related to physical rather than cognitive symptoms.

*Alcohol Use Disorders Identification Test (AUDIT) – C*: We excluded participants scoring 5+, the cut-point indicative of an increasing risk drinker; this was to exclude people in whom memory concerns were directly related to alcohol consumption (Ng Fat et al., 2020).

Participants were invited to be accompanied by a relative/friend (described henceforth as a study partner) in the groups if it facilitated their participation; study partners gave informed consent to participate.

### Interviews and measures

After screening and obtaining written or recorded verbal informed consent, HM conducted baseline assessments – by phone, video-call or prior to lockdown, face-to-face. We recorded sociodemographic characteristics (Table 1), physical disabilities that might restrict participation and screening questionnaire scores (above). An interviewer-administered, semi-structured questionnaire asked participants how the pandemic had influenced: who they spoke to each week, what they ate, their activities, how they accessed help and who provided emotional support or practical help; mental and physical well-being and who they cared for and recording responses in detail. We noted the devices on which they could access groups. We recorded sociodemographic details of study partners.

**Table 1.** Baseline characteristics of participants.

Results are *n* (%) unless specified otherwise	Completed baseline (*n* = 12)	Received intervention (*n* = 10)
Age (years), mean (SD)	74.3 (7.9)	74.3 (8.6)
Gender		
Male	2 (16.7)	1 (10)
Female	10 (83.3)	9 (90)
Ethnicity		
Mixed	1 (8.3)	1 (10)
White	4 (33.3)	3 (30)
Asian	7 (58.3)	6 (60)
Highest education achievement		
Degree or equivalent	8 (66.7)	7 (70)
Higher education	2 (16.7)	1 (10)
Left school after compulsory education	2 (16.7)	2 (20)
Marital status		
Married	2 (16.7)	2 (20)
Divorced	2 (16.7)	1 (10)
Single	4 (33.3)	3 (30)
Widowed	4 (33.3)	4 (40)
First language		
English	4 (33.3)	3 (30)
Other (Cantonese, Sinhala, Philippino, Pujarati and Afrikkana)	8 (66.7)	7 (70)
Employment status		
Retired	11 (91.7)	9 (90)
Full-time	1 (8.3)	1 (10)
Living situation		
Lives with others (with relatives or employer)	6 (50)	6 (60)
Lives alone	6 (50)	4 (40)
Accommodation type		
Owner occupied	5 (41.7)	4 (40)
Lives with employer	1 (8.3)	1 (10)
Council rented	6 (50)	5 (50)
Quick MCI score (mean, SD)	60.2 (7.4)	60.7 (7.3)
Functional assessment score (mean, SD)	3 (3.4)	3.4 (3.6)
AUDIT score (mean, SD)	1.4 (1.3)	1.6 (1.3)

*Data presented represent number (percent) unless otherwise specified. n* = total number of participants with data available; *SD* = standard deviation. MCI: mild cognitive impairment.

Intervention sessions were video-recorded. During goal-setting phone calls (see below), facilitators wrote contemporaneous notes about aids and barriers to achieving goals and recorded participants’ scores on the MDS during sessions 1, 6 and 10. This validated questionnaire is scored from 0 to 16, with higher scores denoting greater Mediterranean-style diet adherence (Valls-Pedret et al., 2015).

Post-intervention, MPo, JBu, CCa and MB conducted semi-structured, virtual qualitative focus groups with intervention participants exploring their experiences; and individual interviews with participants unwilling or unable to attend focus groups, facilitators and study partner(s) (Supplementary Appendix 1: Topic Guides, developed by the study team).

### Analysis

#### Quantitative

We described participants’ sociodemographic characteristics using summary statistics and reported adherence (intervention sessions attended, whether in a planned group, catch-up group or an individual catch-up session) and MDS scores.

#### Fidelity of intervention delivery

Two researchers independently applied checklists to one of the two recorded groups for each of the 10 sessions (after removal of any sessions that failed to record), selected using random number generation (random.org) by the trial manager. We calculated the proportion of expected intervention components (Figure 1) delivered. We adopted established thresholds to rate fidelity (Noell et al., 2002): 81–100% constituted high fidelity, 51–80% moderate and <50% low fidelity. We noted where individual participants did not receive intervention components, and the reason (e.g. connectivity issues and bathroom break). The researchers discussed any discrepancies in ratings, to attain agreement. We reported the mean proportion of intervention components delivered and received by participants, across assessed sessions. We rated on a 5-point scale (1- not at all to 5- very much) whether the facilitator kept the group focused on the manual, and participant(s) engaged, for each intervention component, and for each session, whether the facilitators kept to time.

#### Qualitative

We analysed data collected (1) before the intervention, to provide context, (2) during goal-setting phone calls and (3) post-intervention focus groups and interviews.

##### Content analyses

We carried out content analyses in which two authors (CCo, MPa or JBu) independently evaluated: (1) the extent to which responses to pre-intervention semi-structured questionnaires about how lifestyle and routines had been affected by the Covid-19 pandemic predominantly indicated a negative, positive or neutral/equivocal impact; (2) the types of goals set during goal-setting phone calls, and the aids and barriers participants noted to attaining them.

##### Thematic analysis

We used NVivo12 software to organise data, taking an inductive, adapted thematic analytic approach (Braun & Clarke, 2006). Co-authors (JBu, MPa, CCo, PR, MPo, MB, JBr and NS) systematically and independently double-coded the three sources of qualitative data, initially analysing each source separately. Researchers read texts for accuracy, anonymity and to familiarise themselves with the data, then labelled meaningful fragments of text with initial codes. Discrepancies were discussed by researchers, until a consensus was reached.

**Figure 2.**
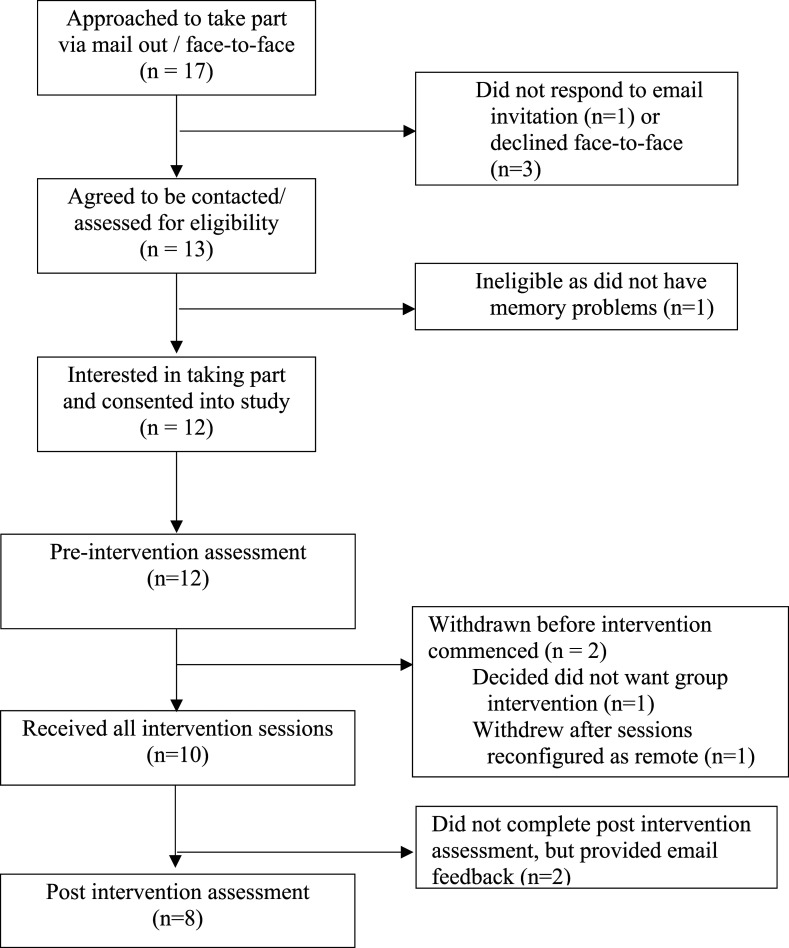
Flow chart for the APPLE-Tree pilot study.

We met as a group to discuss preliminary codes emerging from the data sources and to begin to organise them into preliminary themes addressing research objectives, including to investigate how acceptable and feasible the intervention was to deliver in practice, in the context of the pandemic. We drew on the ‘following a thread’ methodology to iteratively integrate findings from the three data sources, exploring how codes from one dataset followed into the other, and vice versa, developing one interwoven framework (Moran-Ellis et al, 2006). We did not prejudice findings from one data source over another as they provided different insights into the intervention process that we considered equally valid, although most material analysed and reported, stemmed from post-intervention interviews (Figure 2).

## Results

### Recruitment and retention

Twelve of 17 participants approached were eligible, agreed to participate and completed baseline assessments (Figure 1: Flow diagram); four completed baseline assessments in March. One participant withdrew before, and one after being informed in April of plans to shift to remote delivery; the withdrawal after related to a preference for face-to-face groups. Eight further baseline assessments were conducted in June. The semi-structured interview about the impact of Covid was added as an amendment to the design in June and completed by the 10 participants who remained in the study. 10/12 participants completing baseline assessments participated in the intervention and attended post-intervention interviews (*n* = 1) or focus groups (*n* = 5, *n* = 2) or declined to participate in either but sent email feedback (*n* = 2).

### Sample description

Table 1 shows sociodemographic characteristics. Three participants scored >62 on the Quick MCI and met criteria for SCD; and seven met criteria for MCI. Three participants reported hearing loss, and two reported visual impairment that may have interfered with participation.

### Intervention adherence

Groups occurred over 10 weeks in July–September 2020. Two cohabiting participants took part using the same computer. Only 3/10 participants had used Zoom™ before. HM held 10-min practice sessions with all but one participant (who did not need this), before the first group, to explain how to enter the room and use the mute/video buttons. Two participants also required telephone support at the beginning of groups to help them log in. Three participants required technological help throughout the sessions, for example, returning to the correct screen format after viewing videos. One participant involved a study partner – a non-resident daughter, who set up the call and joined the groups.

Table 2 describes attendances and reasons for non-attendance. 92/120 (76.7%) of all possible main group sessions (i.e. for 12 participants completing baseline assessments) were attended or 100/120 (83.3%) including individual catch-up sessions. In addition to the planned sessions, we held one additional catch-up group (for four people) and a total of eight individual catch-up sessions. 77/120 (64.2%) possible refreshment breaks were attended: five participants attended 10; four attended 5–9 refreshment breaks and one participant only joined the final break. Individual goal phone calls took place at each of the 10 time points for all 10 participants attending the intervention. Participants achieved 140/170 (82.4%) of lifestyle goals set (further details in Supplementary Appendix 2).

**Table 2.** Description of participants and APPLE-Tree intervention attendance at each of the 10 sessions (and reasons for non-attendance at group sessions) and post-intervention focus group.

Session	Participant	1	2	3	4	5	6	7	8	9	10	Post-intervention
P1	Female, MCI	UG/T	UG/T	UG/T	UG/T	UG/T	UG/T	UG/T	UG/T	UG/T	ICU(B)/T	Focus group
P2	Male, MCI	UG/T	UG	UG/T	UG/T	UG/T	ICU(A)	UG	CG	UG	UG/T	Individual
P3	Female, MCI	UG/T	UG/T	UG/T	UG/T	UG/T	UG/T	UG/T	UG/T	UG/T	UG/T	Focus group
P4	Female, MCI	UG/T	UG/T	UG/T	UG/T	UG/T	UG/T	UG/T	CG	UG/T	UG/T	Focus group
P5	Female, MCI	UG/T	UG/T	UG/T	UG/T	UG/T	UG/T	UG/T	UG/T	UG/T	UG/T	No
P6	Female, MCI	UG/T	UG/T	UG/T	UG/T	UG/T	UG/T	UG/T	UG/T	UG/T	UG/T	Focus group
P7	Female, MCI	UG/T	UG/T	UG/T	UG/T	UG/T	UG/T	UG/T	UG/T	UG/T	UG/T	No
P8	Female, SCD	ICU (D)	UG	UG	UG	UG	UG	ICU (C)	CG	UG	UG/T	Individual
P9	Female, SCD	UG/T	UG	UG	UG/T	UG/T	UG	ICU (E)	CG	UG/T	UG/T	Focus group
P10	Female, MCI	UG/T	UG	UG/T	UG/T	UG/T	ICU (F)/T	UG	ICU (F)/T	ICU (G)	UG/T	Focus group

P1–P10 denote the 10 participants attending the intervention; in addition two participants recruited at baseline declined attendance at any intervention activities.

UG = attended usual group; CG = attended catch up group (one catch-up group held for session 8); ICU = individual catch-up session received; T= also attended tea break associated with that session.

Reasons for missing group session and requiring individual catch-up: A = family carer unavailable to support with use of Zoom (*n* = 1); B = participant had another competing commitment (*n* = 1); C = feeling anxious about a family event (*n* = 1); D = medical appointment (*n* = 1); E = holiday (*n* = 1); F = work commitment (*n* = 2); G = illness (*n* = 1).

SCD: subjective cognitive decline (Quick MCI score >62; MCI: mild cognitive impairment (Quick MCI score ≤62).

### Fidelity

Overall fidelity (86%) was in the range specified a priori to be high. Mean fidelity scores across intervention components we intended to deliver were assessed as: 4.5 (range 3–5) for ‘keeping the group focussed on the manual/task’; 4.7 (range 3–5) for ‘keeping participants engaged’ and 4.1 (range 3–5) for ‘keeping the session to time’. 23/165 (14%) of components were fully/partially missed by attendees, primarily due to problems with connectivity (assessed for recordings of sessions 2–10 as session 1 recordings were audio, from which continuous presence could not be discerned).

### Thematic analysis: Social connectedness

**Figure 3.**
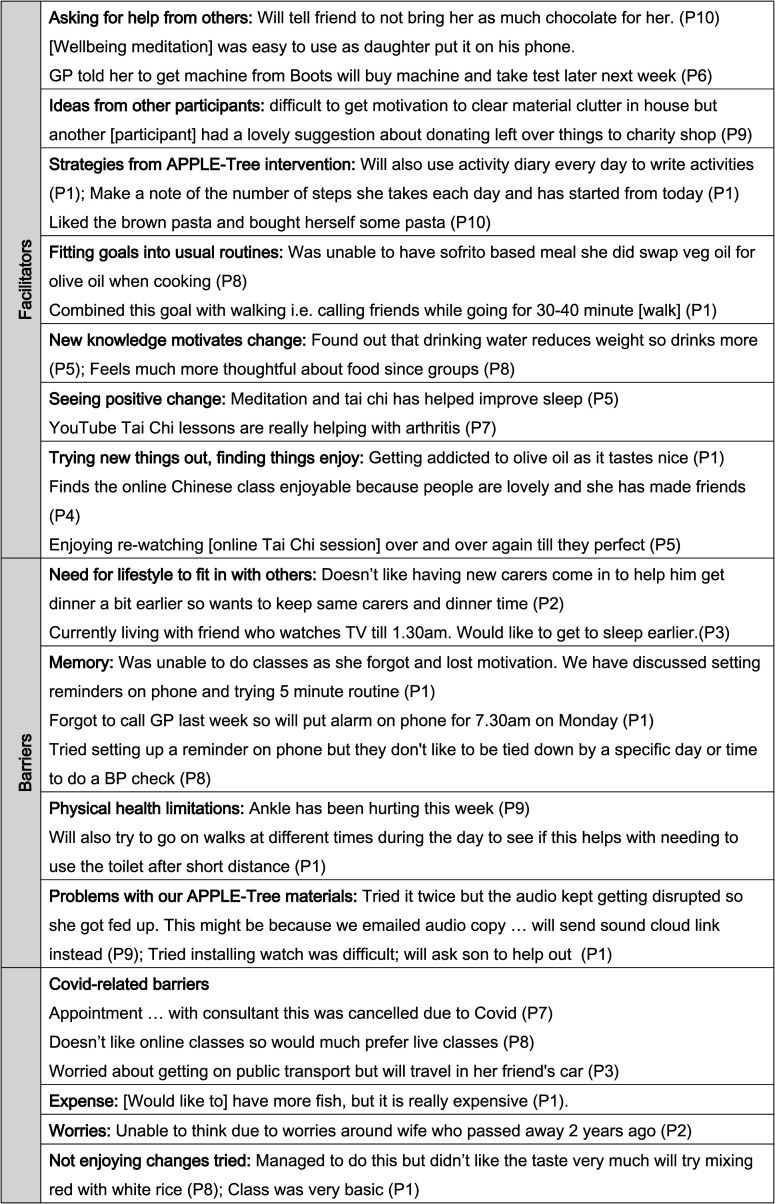
Examples of anticipated facilitators and barriers recorded in goal conversations.

We identified **social connectedness** as an overarching theme, across the three qualitative data sources: pre-intervention interviews (PRE), goal-setting facilitator notes (FN, also listed in Figure 3) and post-intervention focus groups and interviews (POST). We present these findings, noting the source and relevant quantitative data regarding adherence and participant characteristics, which are listed by participant in Table 2.

We describe our theme of social connectedness, with reference to three sub-themes below: (I) **Retaining independence and social connectedness** (social connectedness could not be at the expense of independence); (II) **Adapting social connectedness in the face of the pandemic** (participants strived to compensate for previous social connectedness, as the pandemic reduced support networks) and (III) **Managing social connections within and through the group intervention** (although there were tensions for some participants, they enjoyed social aspects of the groups, which for most were an introduction to the video-call modality. Social connections supported both group attendance and implementation of lifestyle changes, through helping participants to overcome barriers to change, including memory concerns).

## Subtheme I: Retaining independence and social connectedness

It was clear from participants’ accounts that social connectedness was important to them but could not be at the expense of independence. There was a sense that demonstrating independent and resilient behaviours, including providing support to others and adoption of healthier lifestyles, could be reassuring, and a means of distinguishing memory concerns experienced from any intimations of dementia.

While most participants had objective cognitive impairments, and all experienced memory loss, there was a strong sense of independence and resilience in their accounts. Participants described (PRE) providing support to others, including family and friends paying clients and the wider community. For one participant, community work was a major focus; this included ‘*taking a blind person out for guided walks and is involved with local activities at the church’. (P9, PRE*).

This next quote illustrates the sometimes complex interplay between supporting and being supported: a participant described being supported by her friend, while making adjustments to her life to accommodate her friend’s worries:

*[P3, PRE] “is living with her friend who is able to go and get shopping for her. They have also been using online deliveries to get food. Friend was more worried about Covid so participant was unable to go out as much as she would have liked in order to respect her friend’s wishes.”*

Wishes to retain independence and avoid burdening family and friends were predominant sentiments around negotiating support. One participant declined help from neighbours because ‘*she tries to remain independent and do things on her own’. (P4, PRE),* while another felt her daughter was *‘already busy enough to check in on her regularly’ (P9, PRE).*

There was a sense that activity and social contact reassured participants that independence and resilience could be retained. One benefit of attending the APPLE-Tree groups seemed to be the opportunity to demonstrate independence to oneself and the group. This was seen in the context of photo sharing (facilitators showing slides with pictures of crafts or food the group sent to them); these seemed to represent tangible evidence of continued capability, as described by one participant: ‘*just projecting those pictures was … kind of positive reassurance’. (P9, POST)*.

This sense of reassurance was not universal. One participant, who had SCD and attended all groups, but only the final tea break experienced the photo-sharing as ‘*a bit competitive, you know, pictures of people’s beautiful pies and stuff….’. (P8, POST*).

For the helper who attended groups (CCa), the immediacy with which photos of achievements discussed could be shared ‘*potentially add[ed.] to both the positive and negative effects’* described here*.*

Various health or social-related issues were projected as barriers to lifestyle change (Figure 3), which appeared difficult for individuals to circumvent alone. For example, P2 needed the help of his family to renegotiate his care package if he was to be able to achieve his goal to go for a morning walk more regularly: ‘normally goes for a walk in the morning but this is difficult because he does not get dressed until the carers come round in the morning (FN)’. Despite this, change itself was positioned an individual choice and responsibility, with participant P9, who has SCD (POST) describing the groups as:

*“Being kicked up the backside, in a way, to look at oneself all over again and to re-evaluate what we are doing at our age, you know this time of life when we really have to say to ourselves that, “OK, you’re old, but it doesn’t mean to say it’s the end of your life.”*

Adopting individualistic approaches to dementia prevention may have fulfilled an emotional need to distance oneself from intimations of dementia. This could be inferred from this next quote, which also illustrates the reassurance provided by peer support:

*“Somehow, there’s just a reassurance for us people who live alone that maybe we are not going mad and that maybe other people also have memory losses like us, which does not necessarily mean Alzheimer’s.”* P9 (POST)

This illustrates the central tenet of this subtheme, that dementia prevention is best supported by a social connectedness that supports continuation with life despite memory loss and is reassuring rather than one that appears to herald dementia that would be anxiety-provoking and disabling.

### Subtheme II: Adapting social connectedness in the face of the pandemic

Participants described how they strived to maintain social connectedness, as the pandemic reduced support networks, with new arrangements compensating for suspension of face to face activities and services. One person commented that face-to-face contact now only happened ‘*by chance’* (P7, PRE); another that he did not ‘*go out for food as much and has less family gatherings’* (P2, PRE) and another ‘*used to look after her grandchildren but can no longer do this due to lockdown’* (P4, PRE). The pandemic also changed social encounters, even very brief encounters in the community. One participant ‘*has stopped going out for walks as she does not like people looking at her if she wears a mask’* (P6, PRE).

For many participants, the online modality could not entirely compensate for the loss of face-to-face activities, although a minority discovered new connections in the disruption of previous routines. For example, compensatory activities discussed spanned face to face and online modalities, including a group exercise class held by a neighbour on the street and attendance at Vatican Mass online in place of local church attendance. Participant P1 who was recently retired, described how ‘*using more telephone and Zoom meeting … helped widen her social network.’ (PRE).*

We note that P1 was the only participant who did not require facilitator support to access the video-call groups; for most others, the APPLE-Tree groups were their most sustained experience of using video-call and thus of social connections online. P3 (POST), who lived alone, referenced the particular value of the groups as an opportunity for social connection during the pandemic:

*“especially during this Covid time when you couldn’t go out. So, we were able to communicate with each other and looking at each other, and I think that was very good.”*

## Subtheme III: Managing social connections within and through the group intervention

Following on from the previous subtheme, the opportunity for social contact groups provided appeared to be an important reason for the good attendance rates and also for their success in enabling lifestyle change. As P3 (POST), who had MCI and attended all the sessions (Table 2) commented ‘*because we have learned all these things through discussions and connecting to each other, I think we will not forget it’.*

The group planned to continue meeting after the end of the sessions, as noted by P4 (POST), who had MCI and attended all groups and most tea breaks:

*“ [facilitator] did encourage us to form a WhatsApp™ group and then we can still connect together, and we maybe can help each other.”* (P4, POST)

Video calling was a qualitatively different modality for social connections, which was experienced as more distant, and less textured and adaptable than face-to-face contact, although also welcome and novel. P9 [POST] commented ‘*we still get to know each other’s personalities through [video-call] and we don’t have to put on pyjamas or whatever underneath’.*

Facilitators sometimes struggled to address the needs of people who needed more support, within the video-call groups that did not allow for conversations separate from the group.

*‘‘Everybody is in front of you and you are saying that it is sort of a bit upsetting maybe, I did not want to hurt their feelings. Whereas if it is on the side of a table 1 can say “we can talk about that a bit later” quietly so they do not feel like everybody has heard.’ (Facilitator 2/POSTI)*.

This was illustrated from the participant’s perspective by P8, who felt a prevailing positive atmosphere left no space to express other emotions:

*‘‘It was quite nice to listen to other people, but it was all very positive. Nobody ever said “I feel like a lump of shit today” or anything. Nothing like that in it at all. It was all a bit if you weren’t positive you felt you couldn’t say anything”*. (P8, POST)

The differences from face-to-face contact were exemplified by a challenging dynamic created by two participants sharing a device as they were able to talk to each other, while others could not. P8 described how they were ‘*yacking away in their room … You couldn’t hear what anyone else was saying’*.

Goal phone calls were able to compensate for this aspect of the main groups, providing, Facilitator 2 noted, an opportunity for personalisation of the intervention:

*“[Goal phone calls] showed we really cared, and the people noticed that”.*

Social contacts supported the intervention. For example, memory concerns were barriers to lifestyle change that participants often overcame with support from their social networks (FN: Table 3). Forgetting health appointments and social arrangements were of concern to participants, and involving others was one strategy adopted to address these that were often successful. For example, one facilitator recorded that a participant: ‘*finds it difficult to remember to [do relaxation* exercises*] every night and* [his] *daughter will remind him when she calls him before bed’*. (P2/FN). This strategy required support from the participant’s network but also promoted independence.

For one participant, as described in the FN below, compensatory memory strategies suggested in the group that did not involve a relational approach (relying instead on technology) felt too unacceptable and compromising of independence to adopt:

*‘‘Didn’t have time to take blood pressure; tried setting up a reminder on phone but they don’t like to be tied down by a specific day or time to do a BP check’’ (P8, FN)*

Both facilitators interviewed reflected on their experiences of co-facilitating the group remotely. The facilitator who was employed by the third sector organisation felt less connected to the organisation of the groups than the university facilitator, commenting: *[Facilitator 1] was really supportive…. He has a lot to do with the project and I had nothing to do with the project.’*

The third sector facilitators relative lack of familiarity with video calling appeared to contribute to this sense of being a relative outsider, although this also reflected the realities of her employment.

## Discussion

In this pilot trial, adherence to the intervention and fidelity of delivery were high, indicating that it was acceptable and feasible to deliver in practice, even during the Covid-19 pandemic, which did not prevent participants meeting most of the goals they set. For most participants, the groups were a first experience of video calling, so participation directly supported social connectedness during social distancing. Qualitative data indicated that most participants valued the social aspects of the intervention and felt supported by it to make lifestyle changes.

In the thematic analysis, our overarching theme was social connectedness. Three sub-themes gave different perspectives on our central argument that dementia prevention is a social phenomenon, as well as an individual concern. We described how participants negotiated social connectedness while retaining valued independence. Demonstrating independent and resilient behaviours, including providing support to others and adopting healthier lifestyles, was reassuring, a means of distinguishing the memory concerns experienced from intimations of dementia. We explored how participants strived to maintain social connectedness as the pandemic reduced support networks. We describe how the opportunities for social connection groups provided contributed to good attendance; participants and facilitators described the video-call modality as enabling contact, although sometimes as restricting, with one-to-one communication needing to wait for individual goal phone calls. Memory problems and other barriers to changes the intervention targeted were often successfully overcome within relationships.

Living with memory problems can be experienced as a liminal state between wellness and dementia, which medicalises memory concerns yet situates responsibilities for their management with patients and families (Poppe et al., 2020). Our findings that lifestyle change was attainable but often needed support from others, accord with discourses that criticise such individualistic approaches to risk reduction and advocate a social and community psychology of resilience (Cowen, 1994). This reflects concerns regarding the valorisation of agency in contemporary health and social policy (Higgs, 2015).

There was initially some discomfort in our coproduction group that delivering wellbeing groups to older people in a pandemic, which reduces life expectancy (Marois et al., 2020), might seem irrelevant, insensitive, or exacerbate immediate and existential worries. In practice, the intervention was acceptable and feasible to deliver, but these concerns are important to reflect on. Perhaps they represent an attitudinal shift within the team during this period, from individual to societal responsibility for prevention, mirroring reduced individual freedoms around lifestyle and healthcare access during this pandemic. Community-based interventions which promote social support may help create a space for secondary dementia prevention that neither medicalises nor negates the central role of relationships in enabling change. We designed the APPLE-Tree intervention groups for co-facilitation by trained and supervised, non-clinical psychology graduates and community workers. This delivery mode worked well and the intervention was experienced as helpful. Our pragmatic approach mirrors calls in a recent Canadian report, for an integrated approach to later life dementia prevention, which addresses multiple, proximal risk factors, is cost-effective and priced so as to be widely available (Rockwood et al., 2020).

## Limitations

Participants were interviewed immediately post-intervention, so we do not know how changes were sustained, or if memory was impacted over time. Participants may have been more socially connected than those declining participation. Older people are less likely than younger people to use the internet regularly (ONS, 2019); so video-call interventions potentially exclude many older people and could compound existing inequalities. Although most participants were new to video calling, they all had access to devices, and all but one used a device regularly for other purposes. Our current APPLE-Tree trial will evaluate whether our intervention can improve cognition relative to a control group over 2 years. We will, in addition to video-calls, offer face-to-face groups when possible and will loan devices to those without online access. As remote interventions are preferred by some, this blended approach may become standard for future psychological interventions.

## Conclusions

Our intervention was acceptable and feasible to deliver by video-call. Increasing awareness that lifestyle change can help memory could be beneficial at a population level. For more vulnerable populations, such messages need to be communicated alongside supportive, relational approaches to enabling lifestyle changes. The APPLE-Tree intervention manualises such an approach. We commenced an effectiveness trial of the intervention in October 2020 (due to complete 2024). Currently it is delivered remotely, as in the pilot, although when social distancing guidelines allow, we plan to introduce blended remote/face-to-face delivery. If proven effective, this flexible delivery modality is likely to be highly suitable to delivering to populations at scale.

## Supplemental Material

sj-pdf-1-dem-10.1177_14713012211014382 – Supplemental Material for Social connectedness and dementia prevention: Pilot of the APPLE-Tree video-call intervention during the Covid-19 pandemic Click here for additional data file. Supplemental Material, sj-pdf-1-dem-10.1177_14713012211014382 for Social connectedness and dementia prevention: Pilot of the APPLE-Tree video-call intervention during the Covid-19 pandemic by Claudia Cooper, Hassan Mansour, Christine Carter, Penny Rapaport, Sarah Morgan-Trimmer, Natalie L Marchant, Michaela Poppe, Paul Higgs, Janine Brierley, Noa Solomon, Jessica Budgett, Megan Bird, Kate Walters, Julie Barber, Jennifer Wenborn, Iain A Lang, Jonathan Huntley, Karen Ritchie, Helen C Kales, Henry Brodaty, Elisa Aguirre, Anna Betz and Marina Palomo in Dementia
