# Supplementary material for: Social connectedness and dementia prevention: Pilot of the APPLE-Tree video-call intervention during the Covid-19 pandemic
Source: Dementia (London). 2021 Apr 29;20(8):2779–801. doi: 10.1177/14713012211014382 (PMC8679164; doi:10.1177/14713012211014382)
Supplement: sj-pdf-1-dem-10.1177_14713012211014382 – Supplemental Material for Social connectedness and dementia prevention: Pilot of the APPLE-Tree video-call intervention during the Covid-19 pandemic [file sj-pdf-1-dem-10.1177_14713012211014382.pdf]

Appendix 1: Sample topic guide

APPLE-Tree study

Attendee Focus Group Topic Guide

Introductions: Thank you for agreeing to take part in this focus group. As you know I am a researcher from University College London and I will be conducting and recording this focus group. Everything you say is confidential but I would like you to introduce yourselves for the recording so that the typist can identify you. My name is….

Description of the research: You have been invited to attend 10 group APPLE-Tree sessions over the past six months. You may also have received some individual catch-up sessions. I want to ask you about your experiences the sessions you attended, and if you did not attend all of them, what would have encouraged you to do so. We will use your suggestions to develop the intervention manual and training for facilitators delivering it in future, and to understand, if it works or does not work, why this is. There are no right or wrong answers. We would particularly welcome any thoughts about things that could be done differently and really encourage you to be as honest as possible as this is what will help us most.

Q. What did you think about the sessions in general?

Prompts:

- *What did you like best?*
- *What did you find most useful?*
- *What changes were you able to make as a result of the sessions?*
- *Was there anything you did not like?*
- *Was there anything important that you feel was missing?*
- *Were the sessions easy to understand?*
- *What would you like to see changed if anything?*

Q. What did you think about the content of the sessions?

Prompts:

- *What did you like best about the content?*
- *Which topics did you find most useful?*
- *How did the content help you to make changes?*
- *Were there any topics that you did not like?*
- *Were there any important topics that you feel were missing?*
- *Was the content easy to understand?*
- *What would you like to see changed about the content if anything?*

Q. What did you think about the structure of the sessions?

Prompts:

- *What did you think about the number of sessions offered?*
- *What did you think about the length of each session?*
- *What did you think about the length of the overall intervention (3 months)?*
- *How did you find the experience of coming to the groups?*
- *(If any sessions missed): is there anything that might have encouraged you or helped you to attend more sessions?*
- *(if applicable): how did you find the individual catch-up sessions?*
- *What did you think about trying things out/ making changes between sessions?*

- *Is there anything you would like to change about the structure of the sessions?*

Q. Do you have any feedback regarding the layout or design of the materials?
- *How did you find the layout of the materials?*
- *Were the materials easy to read and follow e.g. amount of text on the page, the font, the colours?*
- *What did you think of the videos? Would you like to see more, fewer, anything different?*

How did you find setting up the technology to access the sessions?
How did you find the tea breaks?
As lockdown eases, we may be able to offer groups face-to-face or over zoom. What would you have preferred if you had the choice before the groups? Did your experience change this? What do you think is good about running the groups online? Are there things that might have been better/easier if the groups were face-to-face? Are there things that would have been harder?

Q To what extent do you think the intervention could be useful to you in the future, now that the research study has ended?

Prompts:
- *What would help you use it in the future?*
- *What might stop you from using it in the future?*

Q. Before we finish, is there anything else you would like to mention that we have not already covered?

Thank you for your time and for taking part today.

Appendix 2: Information about goals set by participants during sessions

Goals set included 34/41 (83%) of goals set to increase **Mediterranean-style dietary adherence**. Mediterranean diet scores recorded during sessions 1, 6 and 10; average scores were 10.5 (standard deviation (SD) 3.5); 12.8 (SD 2.8) and 12.7 (SD 3.1) respectively (n=10).

39/45 goals around **physical activity** were met, most commonly around walking, or increasing step counts (8 participants), online exercise videos (4 participants), cycling (3 participants), dancing (5 participants) or Tai Chi (3 participants). In the domain **engaging with life** (see method), 9 participants set at least one goal (range 0-6); 24/30 (80%) goals set were achieved. Topics most frequently covered were: decluttering the house (3 participants), artistic activities (n=3), being in nature (n=3), cooking (n=3), and reading (n=3). 16/17 (96%) goals in the **connecting with others** domain were met. These related to engaging in person (n=15) and online (n=2), and meeting with friends or family (n=14) or new people (n=3).

8/12 (67%) of **health** goals were met, with blood pressure (6 participants), cholesterol (4 participants) and blood glucose (3 participants) checks by a health professional the most common focus. Three participants set goals around increasing fluid intake (100% met). No participants set goals around reducing / managing alcohol use or smoking (no attendees smoked or reported drinking to excess). Eight participants set 13 goals to use relaxation techniques regularly (some breathing, some meditation), of which 10 (77%) were met. 6/9 (67%) of sleep goals, set by 5 participants, were met: most frequent sleep goals were avoiding drinking after 7pm (n=2) and going to bed earlier (n=2).

After session 10, facilitators discussed with participants the changes they wished to continue. All participants identified a physical activity goal; and all but one a dietary goal. Four participants wanted to continue with meditation and breathing goals; three with goals set around “engaging with life”, one with a “connecting with others” goal, one with a sleep goal and one wanted to reduce worry about the future (through mindfulness).
